# Fibrous dysplasia of the sphenoid sinus: headache relieved by surgical excision—a case report

**DOI:** 10.1093/jscr/rjac518

**Published:** 2022-11-19

**Authors:** Jamal Jawad, Derar Al-Domaidat, Mohamed Abaza

**Affiliations:** Otolaryngology Department, Dr. Soliman Fakeeh Hospital, Jeddah, Saudi Arabia; Otolaryngology Department, Dr. Soliman Fakeeh Hospital, Jeddah, Saudi Arabia; Pathology Department, Dr. Soliman Fakeeh Hospital, Jeddah, Saudi Arabia

**Keywords:** Key clinical pointsHeadache, Fibrous dysplasia of sphenoid sinus, surgical intervesion

## Abstract

Fibrous dysplasia (FD) is an uncommon benign bone disorder of unknown etiology in which normal medullary bone is replaced by fibrotic and osseous tissue. FD of paranasal sinuses is usually secondary to extension from adjacent bones. It is rarely limited to the sinuses, let alone limited to the sphenoid sinus. Furthermore, the relationship between headache and FD of paranasal sinuses has not been well addressed. We report a case of a 55-year-old female with FD of sphenoid sinuses complaining of sever right-sided parietal headache. Her headache was significantly improved after surgical excision of FD.

## INTRODUCTION

FD is a localized disorder of bone characterized by abnormal proliferation of fibrous tissue interspersed with normal or immature bone. Three general subtypes of disease are recognized: monostotic, polyostotic and McCune-Albright syndrome [1, 2]. The skull and facial bones are affected in 10–25% of patients with monostotic subtype and in 50% of patients with polyostotic subtype [3]. FD shows a tendency for facial and cranial bones most frequently located in the mandible and maxilla. Paranasal sinuses are rarely involved. FD has a potential to cause deformity and dysfunction in the facial and cranial bones [4]. Atypical facial pain and headache are the most common presenting symptoms, followed by symptoms of sinusitis. Other reported symptoms and signs include: visual changes, proptosis, diplopia, orbital pain or pressure and facial numbness [1]. Although it is a rare disease entity and it is not enough to know about the causal relationship between FD of the sphenoid sinus and headache, we should keep in mind the headache is the most common symptom among a wide spectrum of potential symptoms in craniofacial FD [5]. In this paper, we report a rare case of FD of the sphenoid sinus presented with severe unilateral headache, which was successfully treated with excision using endoscopic transeptal approach to sphenoid sinuses.

## CASE PRESENTATION

**Figure 1:**
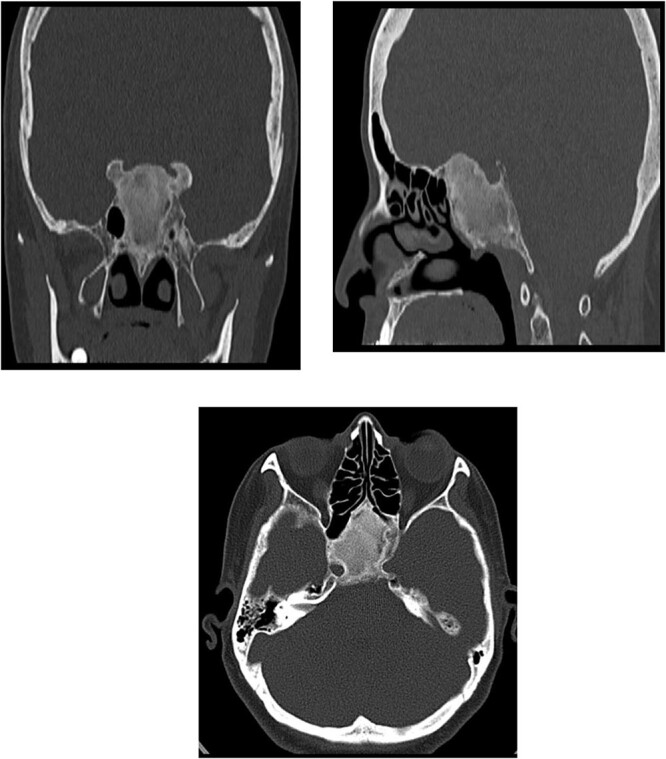
CT of the paranasal sinuses showing expansion of the sphenoid bone demonstrating diffuse ground-glass matrix and sclerosis obliterating the sphenoid sinus cavity.

**Figure 2:**
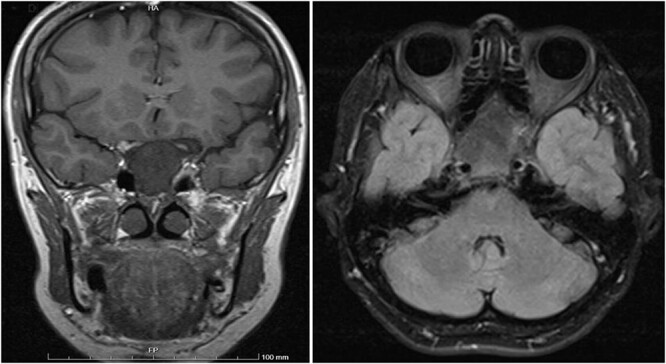
Coronal T1 image showing the expansion of the clivus with heterogeneous predominantly low T1 signal. Axial flair images showing the anteroposterior (AP) extension of the mass lesion with obliteration of the sphenoid sinuses. The cavernous portions of both internal carotid arteries (ICAs) have signal void intensity suggesting patency.

**Figure 3:**
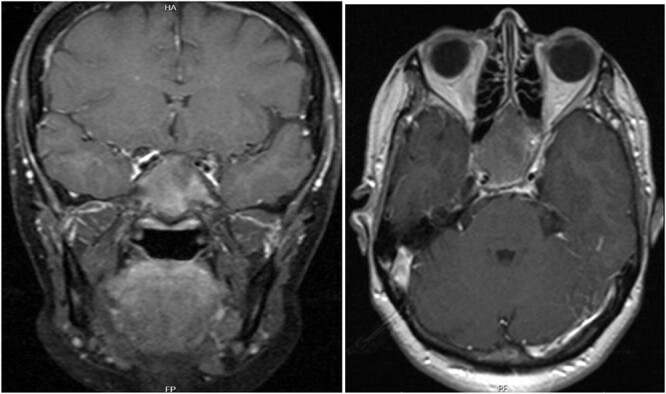
T1 axial and coronal post-contrast images show some heterogeneous enhancement compared to pre contrast images.

**Figure 4:**
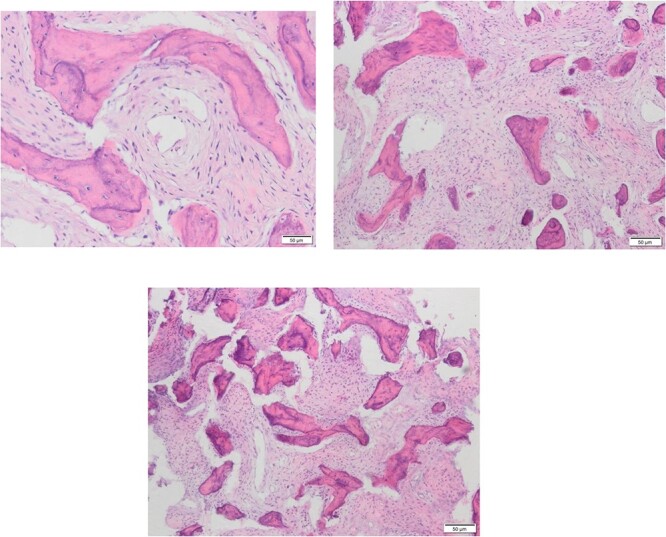
Different Histopathological slides confirming the diagnosis of fibrous dysplasia which presents under microscope as branching irregular trabeculae of woven bone with no osteoblastic rimming. There is intervening hypocellular fibrous stroma.

A 56-year-old female presented with a 1-year history of intermittent headaches, which had been increasing in severity and intensity over the last 2 months. The headache was mainly in the right parietal region, throbbing in nature and did not display diurnal variation. The severity of the headache was stated as 7–8 degrees on the Numerical Rating Pain Scale (NRPS; [6]). The pain was partially relieved with analgesia (acetominophen and ibuprofen). She also complained of mild right nasal congestion and intermittent mild otalgia. Physical examination was normal apart from nasal septal deviation. Computed tomography (CT) of the paranasal sinuses showed expansion of the sphenoid bone demonstrating diffuse ground-glass matrix and sclerosis obliterating the sphenoid sinus cavity (Fig. 1). Magnetic resonance imaging (MRI) was highly suggestive of fibrous dysplasia of the sphenoid bone, which appeared as expanded mass with dark T2 signal and T1 hypointense signal (Figs 2 and 3). Near-total excisional biopsy was performed using endoscopic transeptal approach to sphenoid sinuses. Histopathological examination of several pieces of greyish tan and brown bony tissue measuring 3.5 × 2.5 × 1.7 cm. showed branching irregular trabeculae of woven bone with intervening hypocellular fibrous stroma, consistent with a diagnosis of FD (Fig. 4). The patient’s headaches improved greatly following surgery. Two years post-op she reports infrequent headache with a severity of 2 on NRPS.

## DISCUSSION

The most commonly affected areas of the skull base are; the ethmoid, sphenoid, followed by the frontal bone and maxilla [1]. However, some older literature reviews reported that the order of prevalence of FD is as follows: the frontal bones were most commonly involved, followed by the sphenoid, ethmoid, parietal, temporal and occipital bones [7]. The differential diagnosis of fibrous dysplasia includes: simple bone cysts, non-ossifying fibromas, osteofibrous dysplasia, adamantinoma, low-grade intramedullary osteosarcoma and Paget’s disease [4]. Radiographic patterns of FD have been classified as pagetoid, sclerotic and cyst-like. The pagetoid form resembles Paget’s disease and is seen in 56% of all cases and is characterized by alternating zones of radiodense and radiolucent areas. The sclerotic pattern is homogeneously dense and is seen in 23% of patients and is frequently observed in the monostotic type. The cyst-like pattern is seen in 21% of patients and is characterized by a spherical or oval shape and well-defined radiolucency surrounded by a dense rim, resembling an eggshell [4]. CT is the study of choice for diagnosis and follow-up because of its superior bony detail and accurate assessment of the extent of the lesion. Furthermore, CT can often assist with differentiating fibrous dysplasia from other osteodystrophies of the skull base [1]. CT is also important for surgical planning, and in follow-up for measuring growth rate. A ‘ground-glass-like’ appearance is characteristic of FD [4]. On MRI, FD lesions typically show sharply demarcated borders and intermediate to low signal intensity on T1-WI and intermediate to high intensity on T2-WI. All lesions show some degree of enhancement on post-contrast T1-WI, but the enhancement pattern may be patchy central, rim, homogeneous or a combination. Therefore, MRI is not particularly useful in differentiating FD from other entities [8]. According to the literature, surgery should be reserved for patients with functional impairment or a cosmetic deformity. Because of the benign nature of the condition, the surgery itself should be relatively conservative, with the primary goal being preservation of existing function [1]. Our case demonstrates that surgery to treat FD of the sphenoid sinus can alleviate associated headaches successfully.

## CONCLUSION

Isolated sphenoid sinus FD is rare. Patients with FD of the sphenoid sinus may present with chronic, unilateral, severe headaches. Surgery has historically been reserved for functional and aesthetic deformities, as well as in cases of threat to vital structures. We demonstrate that surgical excision of sphenoid sinus FD can alleviate headaches significantly.
